# Cystatin from Austrelaps superbus snake venom as a model for identifying potential inhibitors of Trypanosoma cruzi cruzain

**DOI:** 10.1590/1678-9199-JVATITD-2024-0055

**Published:** 2025-02-14

**Authors:** Jorge Javier Alfonso Ruiz Díaz, Ana Fidelina Gómez Garay, Anderson Makoto Kayano, Rudson Holanda, Aleff Ferreira Francisco, Christian Collins Kuehn, Andreimar Martins Soares, Celeste Vega, Leonardo de Azevedo Calderon

**Affiliations:** 1 Center for the Development of Scientific Research (CEDIC), Asunción, Paraguay.; 2 Center for the Study of Biomolecules Applied to Health (CEBio), Oswaldo Cruz Foundation (Fiocruz), Fiocruz Rondônia Unit, Porto Velho, RO, Brazil.; 3 International Network for Research and Excellence Knowledge of Western/Eastern Amazon (RED-CONEXAO), Porto Velho, RO, Brazil.; 4 Center for Research in Tropical Medicine (CEPEM/SESAU-RO), Porto Velho, RO, Brazil.; 5 Federal University of Amazonas (UFAM), AM, Brazil.; 6 Laboratory of Biochemistry and Biophysics, Butantan Institute, São Paulo, SP, Brazil.; 7 Department of Medicine, Federal University of Rondônia, Porto Velho, RO, Brazil.; 8 Laboratory of Biotechnology of Proteins and Bioactive Compounds Applied to Health (LABIOPROT), Oswaldo Cruz Foundation (Fiocruz), Fiocruz Rondônia Unit, Porto Velho, RO, Brazil.; 9 National Institute of Science and Technology in Epidemiology of the Western Amazonia (INCT-EpiAmO), Porto Velho, RO, Brazil.; 10 São Lucas Porto Velho University Center, Porto Velho, RO, Brazi.

**Keywords:** Chagas disease, Cruzain, Inhibitors, Snake venom, Cystatins

## Abstract

**Background:**

Chagas disease (CD), caused by *Trypanosoma cruzi*, affects approximately seven million individuals worldwide, with the highest number of cases in Latin America. CD has two phases, of which the chronic phase is characterized by reduced efficacy in drug therapies. This and other factors make developing new strategies that aim to identify molecules capable of becoming alternatives to or complement current chemotherapy vitally important.

**Methods:**

Cruzain and AsCystatin were obtained recombinantly through expression in *E. coli*. Bioinformatic assays were conducted with both molecules, followed by *in vitro* enzyme inhibition assays. Subsequently, *in silico* studies allowed for the design of peptides, which were then assessed for molecular interactions with cruzain. The designed peptides were synthesized, and their inhibitory potential on cruzain and their trypanocidal and cytotoxic effects *in vitro* were finally assessed.

**Results:**

AsCystatin, a potential inhibitor of cysteine proteases, was identified from previously published scientific literature. *In silico* assays suggested that AsCystatin interacts with key regions of cruzain, and was subsequently produced through heterologous expression, obtaining a protein with a high degree of purity. Next, the inhibition of AsCystatin on the activity of cruzain was assessed, observing that approximately 20 µM of cystatin could inhibit 50% of the catalytic activity of the recombinant enzyme. Based on the *in-silico* analysis performed previously, original, and modified peptides were designed and tested, which allowed for identifying four peptides with inhibitory capacity on the enzymatic activity of cruzain. Finally, three of these peptides showed trypanocidal activity on epimastigote forms of *T. cruzi* in *in vitro* models.

**Conclusion:**

It was possible to identify AsCystatin and four peptides derived from this protein with inhibitory activity on cruzain, highlighting the trypanocidal effect of these peptides observed in *in vitro* assays.

## Background

Even though over 110 years have passed since its discovery, Chagas disease (CD), classified by the World Health Organization (WHO) as a neglected tropical disease [1], continues to represent a significant public health problem [2]. According to WHO data, it is estimated that 6 to 7 million people worldwide could be infected with *Trypanosoma cruzi,* the causative agent of this disease [1]. It should be highlighted that American trypanosomiasis is endemic to Latin America and affects vulnerable populations, primarily residents of rural areas with poor living conditions, which facilitates the proliferation of the vector in homes. Despite significant efforts to combat the disease, approximately 75 million individuals remain at risk of infection [1-3].

Clinically, CD is divided into two well-defined phases: (i) the initial phase of infection, known as the acute phase, lasting 4 to 8 weeks, and (ii) the chronic phase, which extends over a long period [4]. It should be noted that in this stage of the disease, the effectiveness of treatment is considerably lower compared to the acute phase, which correlates the chronic phase of the disease with a high mortality rate [5].

Regarding the treatment of CD, it is limited to only two drugs: benznidazole (Rochagan^®^, Roche) and nifurtimox (Lampit^®^, Bayer) [6]. Although it is estimated that approximately 80% of acute cases can achieve parasitological cure with treatment using these drugs, their efficacy during the chronic phase is considerably limited. In addition, other aspects, such as the adverse effects of these drugs and the resistance of various *T. cruzi* strains to these drugs, have made CD chemotherapy even more complicated, highlighting the urgent need for alternative strategies to identify new molecules with potential antiparasitic activity [7, 8].

Given this, various approaches have been explored, including identifying and characterizing essential molecules in infectious agents called pharmacological targets. From this, the search began for agents capable of inhibiting these key targets in parasites, consequently affecting these microorganisms’ metabolism and/or viability [9-11]. In this regard, a widely studied pharmacological target is cruzain, the principal cysteine protease of *T. cruzi*. This protease is associated with various processes such as immune system evasion, proliferation, differentiation, and cellular invasion, making it a key enzyme for the parasite's metabolism [12-15] and the focus of numerous research groups for developing strategies to identify inhibitors of its enzymatic activity [16-18].

Concerning the search for inhibitors of this cysteine protease’s enzymatic activity, synthetic and natural molecules with a wide range of chemical and structural diversity have been identified [16, 19-22]. Various authors have explored cystatins as potential inhibitors of cysteine proteases [23-25]. Considering that snake venoms are described in the scientific literature as a valuable source for identifying molecules with potential antiparasitic activity due to the wide range of biological activities of their components [20, 26-30], this study proposes using a cystatin present in the venom of *Austrelaps superbus* (AsCystatin) as a model to assess its inhibitory potential against cruzain as well as its trypanocidal and cytotoxic activity *in vitro.*

## Methods

To achieve the objectives outlaned in this study, the research was divided into four main stages: (i) heterologous expression of cruzain and AsCystatin proteins, (ii) *in silico* analysis of the interaction between the receptor and its potential ligands, (iii) determination of the *in vitro* inhibition capacity of AsCystatin and derived peptides on cruzain enzymatic activity, and finally (iv) assessment of the *in vitro* trypanocidal and cytotoxic activity of peptides derived from AsCystatin. Each methodology employed is briefly detailed below.

### Plasmid DNA amplification

The vector pET 21a (+) from Novagen containing the pro-cruzain gene was kindly provided by Dr. Maximiliano Juri Ayub from the Multidisciplinary Institute of Biological Research of San Luis, Argentina *(*Instituto Multidisciplinario de Investigaciones Biológicas de San Luis, Argentina*).* Chemically competent *E. coli* TG1 cells were transformed to amplify the genetic material. Plasmid DNA extraction and purification were performed using the miniprep kit (QIAGEN®), following the manufacturer's instructions.

### Expression and purification of pro-cruzain

For the expression of the target molecule, the methodology proposed by Lee et al. [31] was used with some modifications. To a 100 μL culture of chemically competent *E. coli* BL21 DE3 cells, 5 μL of a 40 ng/μL of the pET 21a (+) plasmid was added. The bacteria were incubated on ice for 15 minutes, followed by a thermal shock by placing the mixture of bacteria and plasmid in a thermoblock at 42°C for 90 seconds, then transferring them back to ice for 15 minutes. Finally, 300 μL of SOC culture was added, and the cell suspension was incubated for an hour at 37°C in a shaker at 250 RPM.

After incubation, 100 μL of the culture was placed on a Petri dish with LB medium supplemented with ampicillin at a final 100 μg/mL concentration. The plate was incubated overnight at 37°C, and a colony was selected and inoculated into a conical tube with 5 mL of LB medium supplemented with ampicillin and incubated for 16 hours at 37°C in a shaker at 250 RPM. Subsequently, 2.5 mL of the culture was transferred to a flask with 250 mL of LB medium supplemented with ampicillin, and when the culture reached an OD_600_ of 0.6 AU, IPTG was added to a final concentration of 1 mM. The culture was induced for 72 hours at 18°C with shaking at 250 RPM. After induction, the culture was centrifuged, the supernatant was discarded, and the pellet was stored at -20°C until cell lysis.

### Polyacrylamide gel electrophoresis at 12.5% in the presence of sodium dodecyl sulfate (SDS-PAGE 12.5%)

For this procedure, a pellet from 500 μL of pre- and post-induction culture was incubated with sample buffer at 95°C for 5 minutes and then subjected to SDS-PAGE 12.5% as described by Laemmli [32]. After the procedure, the gel was washed with deionized water and fixed in a 40% methanol and 7% acetic acid aqueous solution for 30 minutes. Subsequently, the gel was stained for 30 minutes with gentle shaking in a PhastGel™ Blue R (GE Healthcare) solution. Excess dye was removed by immersing the gel in a 4% ethanol and 7% acetic acid solution. Finally, the image was documented using LabScan® software (GE Healthcare).

### Cell lysis and purification of pro-cruzain

The pellet from the induced culture was resuspended in 25 mL of lysis buffer and incubated on ice for 2 hours. The material was then subjected to sonication (Sonicator S-4000, Misonix®) in four cycles of 30 seconds with an amplitude of 40 and 30-second intervals. The resulting product was centrifuged at 4000 *xg* for 30 minutes, and the supernatant was filtered through a 0.22 μM membrane (Millex-HV, Millipore®) to remove impurities.

For pro-enzyme purification, immobilized metal affinity chromatography (IMAC) was performed. In this phase, a column (1 × 5) cm with Nickel (Ni-NTA) agarose resin (QIAGEN®) was equilibrated with 10 column volumes of wash buffer (TRIS 50 mM, NaCl 300 mM, and imidazole 10 mM) and then the product from cell lysis was applied. Non-specific proteins were eluted with five-column volumes of wash buffer, and finally, the elution buffer (TRIS 50 mM, NaCl 300 mM, and imidazole 10 mM) was applied. The recombinant protein was eluted in a gradient of elution buffer from 0 to 100%, with a continuous flow of 1 mL/min for 20 minutes. An Akta Purifier chromatograph (GE Healthcare Life Science) was used, and the procedure was monitored at an absorbance of 280 nm. To prevent degradation of the eluted protein, protease inhibitors phenylmethylsulfonyl fluoride (PMSF) and methyl methanethiosulfonate (MMTS) were added to a final concentration of 1 mM. The eluted product was analyzed by SDS-PAGE 12.5%.

### Activation of recombinant protease

For the activation of pro-cruzain, the methodology proposed by Lee et al. [31] was adopted. The eluate from chromatography was dialyzed for 16 hours in activation buffer (sodium acetate 100 mM, pH 5.5, NaCl 300 mM, EDTA 5 mM, and DTT 5 mM). This process was carried out with shaking at 4°C. The activation by auto-proteolysis step was initiated by adding 5 mM DTT and incubating the enzyme at 37°C in a dry bath. For 2 hours, 20 μL samples were withdrawn at specific time intervals to monitor the activation process by SDS-PAGE 12.5%. Once the active protease was obtained, it was stored in 5 mg/mL aliquots at 4°C until use.

###  Homology modeling of Austrelaps superbus cystatin

To create a homology model of the potential inhibitor the NCBI database was searched for sequences homologous to the cystatin of *A. superbus* using the local alignment tool (http://blast.ncbi.nlm.nih.gov/48) (BLAST). Amino acid sequence corresponding to AsCystatin was obtained by Richards et al. [33], whereas human cystatin M (PDB code 4N60), was used as a template. Modeling was performed using the Modeller 9.19 program, and the generated three-dimensional model was validated on the PROCHECK platform [34]. 

### Molecular docking between AsCystatin and cruzain

The generated model was subjected to an in silico molecular interaction assay with cruzain using the ClusPro 2.0 tool [35]. The three-dimensional model of cruzain used is deposited in the Protein Data Bank (PDB, code: 1AIM), and the complex formed by the interaction of both proteins was analyzed using the UCSF Chimera program [36].

### Obtaining the vector with the AsCystatin gene

The synthesis and optimization process of the gene of interest in the expression vector was performed by FastBio® (São Paulo, Brazil). The sequence corresponding to AsCystatin (GenBank code: Fj411278) was optimized and inserted into the expression vector pET28a (+). The vector size was 5369 bp and included a region conferring kanamycin resistance, which is why the culture medium used for bacteria transformed with this vector was supplemented with the mentioned antibiotic at a final concentration of 25 μg/mL.

## Expression of AsCystatin

The methodology proposed by Richards et al. [33] was adopted to express the potential inhibitor with some specific modifications. Initially, the transformation and expansion of *E. coli* BL21 DE3 culture were performed as described in the section “Expression and purification of pro-cruzain”. Induction was carried out in a shaker at 16°C for 16 hours, assessing two concentrations of the inducer IPTG (0.5 and 0.1 mM). The electrophoretic profile of the induced bacteria was assessed by SDS-PAGE 12.5%.

### Cell lysis and purification of AsCystatin

Induced bacteria were subjected to cell lysis by resuspending the pellet in 25 mL of Tris 50 mM, pH 8, and lysozyme (1 mg/mL), and incubated on ice for 1 hour. The mixture was then subjected to sonication and centrifuged, and the resulting supernatant was filtered, as detailed in the section “Cell lysis and purification of pro-cruzain”. Finally, the protein of interest was purified via IMAC chromatography and analyzed by SDS-PAGE 12.5%.

### Assessment of the inhibitory potential of AsCystatin on cruzain enzymatic activity

Cruzain at a final concentration of 50 μM was incubated for 30 minutes at 37°C with various concentrations of AsCystatin (50-6.25 μM). Subsequently, 1% casein substrate diluted in Tris-HCl 0.1 M, pH 8, was added. The mixture was incubated again for 30 minutes at 37°C, and the reaction was stopped by adding 250 μL of 20% Trichloroacetic Acid (TCA), followed by a 30-minute rest period at room temperature. Finally, the mixture was centrifuged at 10,000 × *g* for 15 minutes. Enzymatic activity inhibition was estimated by recording the absorbance of the supernatant at 280 nm using a microplate spectrophotometer (BioTek Eon). The concentration required to inhibit 50% of cruzain enzymatic activity was determined using GraphPad Prism 6.0 software.

### Design of peptides derived from the primary structure of AsCystatin and molecular docking between the peptides and cruzain

Following a detailed analysis of the primary structure of the cystatin and the results obtained from the molecular docking between both proteins, eight peptides derived from the primary structure of the potential inhibitor were designed. Four linear peptides and four mimetic peptides were constructed, named Pep1 to Pep8. For linear peptides, the N-terminal region (Pep1) and the loop 1 and loop 2 regions for Pep2 to Pep4 were considered, whereas for mimetic peptides (Pep5 to Pep8), the three conserved structural elements of cystatin, such as the N-terminal region, loop 1 and loop 2, were considered.

The peptides were subjected to *in silico* molecular interaction assays with cruzain. Simulations were performed using PyRx 9.4 software with AutoDock Vina 1.1.2. Analysis and editing of the formed complexes were carried out using the UCSF Chimera program [36].

### In vitro assessment of the inhibitory potential of peptides derived from AsCystatin on cruzain enzymatic activity

The peptides designed, as mentioned in the previous section, were synthesized by AminoTech (São Paulo, Brazil). Subsequently, the ability of the synthetic peptides to inhibit cruzain enzymatic activity was assessed. For this procedure, different concentrations of each peptide (500 to 62.5 μM) were incubated with 50 μM cruzain, and the steps detailed in the section “Cell lysis and purification of AsCystatin” were carried out.

### Assessment of the in vitro trypanocidal potential of synthetic peptides

To assess the trypanocidal activity of the synthetic peptides, epimastigote forms of clone CL B5 of *Trypanosoma cruzi*, transfected with the *Escherichia coli* β-galactosidase gene (lacZ), were used. The parasites were cultivated at 28°C in liver infusion tryptose (LIT) medium, supplemented with 10% fetal bovine serum (FBS) and the antibiotics penicillin and streptomycin. About 200 µL of epimastigotes at 2.5 × 10^5^ parasites/mL concentration were plated in a 96-well plate. The parasites were incubated at 28°C for 72 hours with different concentrations of peptides (500-62.5 µM). Then, 50 µL of chlorophenol red-β-D-galactopyranoside (CPRG) solution was added, reaching a final concentration of 200 µM. The plate was incubated again at 37°C for 4 hours, and absorbance was monitored at 590 nm using a spectrophotometer (Synergy H1 multi-mode BIOTEK). Benznidazole was used as a positive control. The half maximal inhibitory concentration (IC_50_) was determined using GraphPad Prism (v.6.01) software.

### Assessment of the in vitro cytotoxicity of synthetic peptides

In these experiments, NCTC clone 929 fibroblasts were cultured in a 96-well plate with 180 µL of minimal essential medium (MEM) supplemented with 10% FBS, penicillin and streptomycin antibiotics. About 2 × 10^4^ cells per well were cultured and incubated at 37°C for 48 hours in a 5% CO_2_ environment with different concentrations of synthetic peptides (500-62.5 µM). Then, 20 µL of a resazurin solution was added, reaching a final concentration of 2 mM. The plate was incubated again at 37°C for 4 hours, and absorbance was monitored at 570 and 600 nm using a Synergy H1 multi-mode spectrophotometer (BIOTEK). GraphPad Prism software determined the half-maximal cytotoxic concentration (CC50) (v.6.01). The CC50 of the reference drug benznidazole was also determined to finally determine the selectivity index

## Results

### Expression, purification, and activation of cruzain

**Figure 1.**
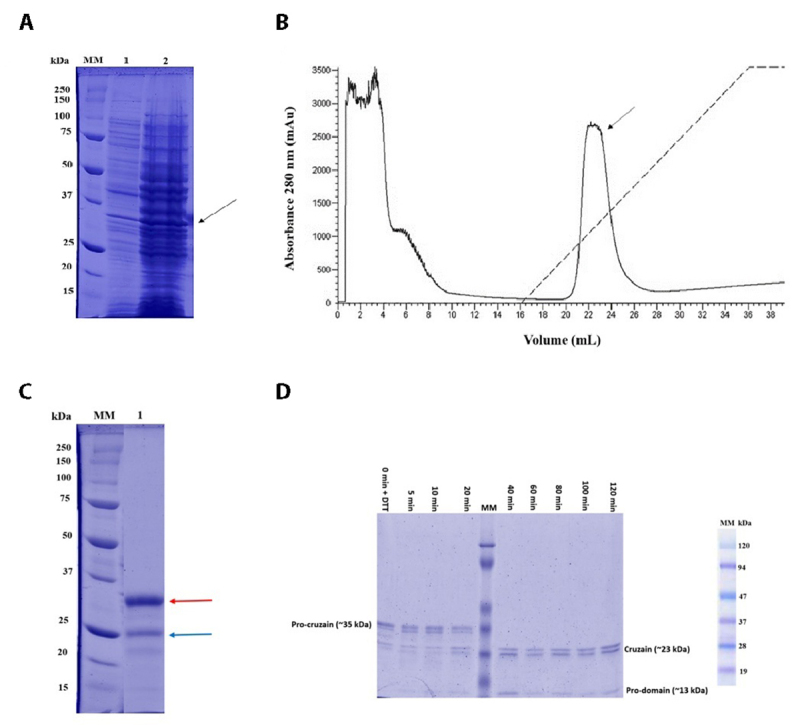
Expression, purification, and activation of cruzain. **(A)** Bacterial lysate before (lane 1) and 72 hours after induction (lane 2). An arrow indicates a band with proteins of approximately 35 kDa, consistent with the molecular mass of pro-cruzain. **(B)** IMAC allowed the purification of the protein of interest, which was eluted with approximately 35% of the elution buffer. **(C)** Electrophoretic profile of the purified protein, showing a main band of approximately 35 kDa (red arrow) and a faint band of approximately 23 kDa (blue arrow). **(D)** Electrophoretic profile of the recombinant protein maturation process. After two hours, pure and enzymatically active cruzain was obtained at an appropriate concentration for further experiments.

*E. coli* BL21 DE3 strains were used to produce recombinant cruzain. Figure 1 A shows the electrophoretic profile of the lysate of transformed bacteria before adding the inducer (lane 1) and 72 hours after IPTG induction (lane 2). A prominent band of approximately 35 kDa, compatible with pro-cruzain, is observed. The lysate was then subjected to affinity chromatography, where the enzyme was purified with a high degree of purity using approximately 35% elution buffer (Figures 1 B and 1C). Finally, cruzain was activated by auto-proteolysis. Figure 1 D shows that the protein is in its zymogen form up to 20 minutes, and from 40 minutes onwards, only the mature protease is visible. A protein band of approximately 13 kDa, corresponding to the pro-domain cleaved by the auto-proteolysis process, is also observed. From 40 to 120 minutes, mature cruzain was obtained in a soluble and enzymatically active form.

### In silico interaction between cruzain and AsCystatin

**Figure 2.**
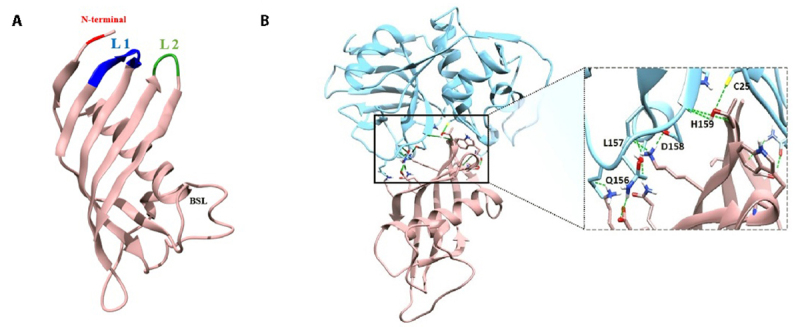
Homology modeling of AsCystatin and *in silico* interaction analysis with cruzain. **(A)** The created model shows that AsCystatin has conserved tertiary structural elements characteristic of group 2a cystatins, such as the "cystatin fold," consisting of five antiparallel β-strands surrounding a central α-helix, as well as loop 1 and loop 2. **(B)** Molecular docking indicates that key sites of cruzain interact with *A. superbus* cystatin, suggesting that this molecule could act as an inhibitor of *T. cruzi* protease.

To determine the *in silico* interaction between the two molecules, homology modeling of cystatin was performed, followed by an interaction assay between the pharmacological target and its potential inhibitor. Figure 2 A represents the generated three-dimensional model, highlighting conserved structural elements of cystatins such as the N-terminal region, loop 1, loop 2, and a region termed back side loop (BSL). 

On the other hand, molecular interaction analysis suggests that key regions of the cysteine protease, such as Cys25 and Hist159, located at the enzyme's active site, interact with the cystatin (Figure 2 B).

### Expression and purification of AsCystatin

**Figure 3.**
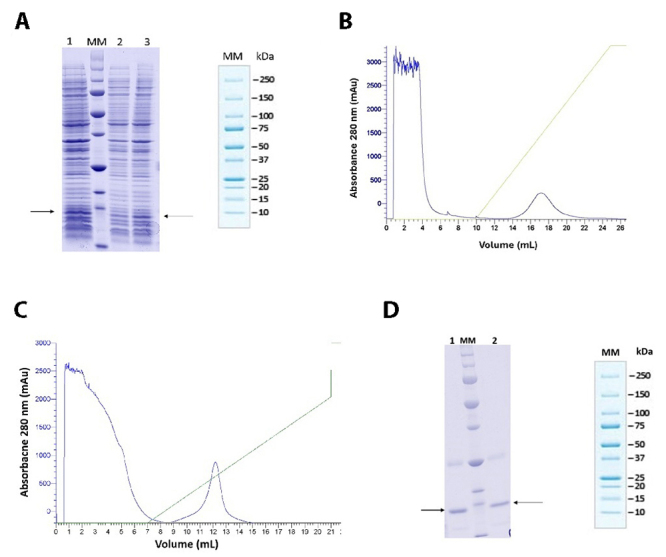
Expression and purification of AsCystatin. **(A)** Electrophoretic profile of bacterial lysate before (lane 2) and 16 hours after induction with 0.1 mM (lane 1) and 0.5 mM (lane 3) IPTG. An arrow indicates a band with proteins approximately 13 kDa, consistent with the molecular mass of AsCystatin. **(B, C)** IMAC allowed the purification of the protein of interest, which was eluted with approximately 30% of the elution buffer. **(D)** Electrophoretic profile of the purified protein, showing a main band of approximately 13 kDa (indicated by an arrow in lanes 1 and 2).

To produce recombinant AsCystatin, the *E. coli* BL21 DE3 strain was used. Figure 3 A shows the electrophoretic profile of the bacterial lysate before (lane 1) and 16 hours after induction with 0.1 mM (lane 2) and 0.5 mM (lane 3) IPTG. Bands with proteins of approximately 13 kDa molecular weight are indicated. Figures 2 B and 2C show the chromatographic profile from the protein purification process. These profiles demonstrate that in both assessed conditions, the protein was eluted with approximately 30% elution buffer, as confirmed by the electrophoretic profile in Figure 3 D, where bands with protein molecular weights compatible with cystatins are observed in lanes 1 and 2.

### Inhibitory effect of AsCystatin on cruzain enzymatic activity

**Figure 4.**
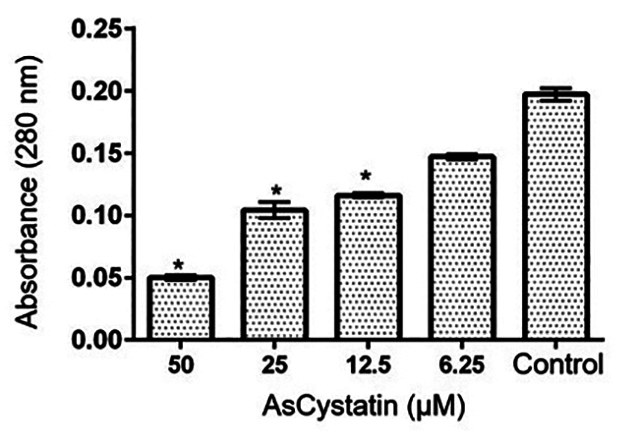
Compared to cruzain activity (control), AsCystatin was able to inhibit the proteolytic activity of cruzain at most concentrations assessed, with a concentration of 21.2 µM required to inhibit 50% of cruzain's enzymatic activity. Statistical analysis was performed using GraphPad Prism6 with ANOVA and Turkey tests. Asterisks indicate significant differences (p < 0.05) compared to the control.

Once both molecules were obtained, an enzymatic inhibition assay was conducted. Figure 4 shows that most assessed concentrations significantly reduced the proteolytic activity of cruzain, with a concentration of 21.2 μM being able to inhibit 50% of its enzymatic activity (Table 1).

**Table 1.** Inhibitory effect of AsCystatin on cruzain enzymatic activity.

Potential cruzain inhibitor	IC_50_ (μM) ± SD
AsCystatin	21.2 ± 0.25

### In silicointeraction between Cruzain and peptides derived from cystatin

**Figure 5.**
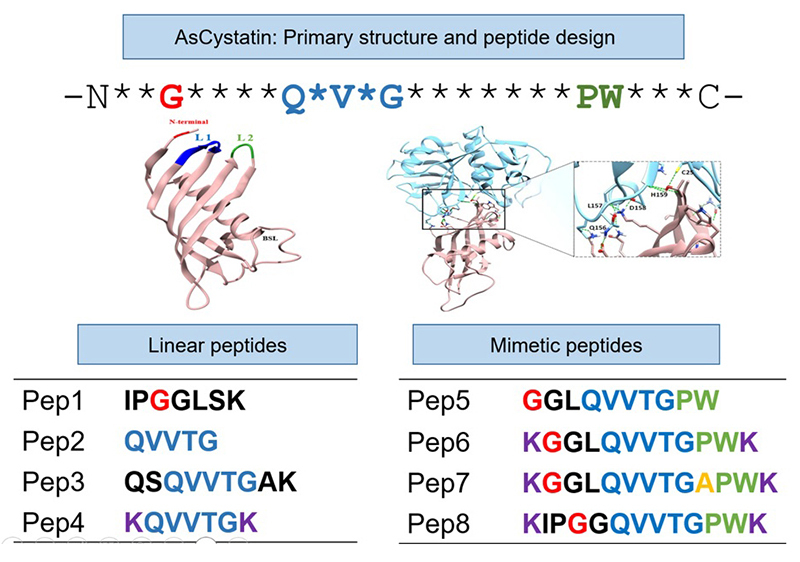
Analysis of the primary structure and the complex formed between cruzain and AsCystatin led to the construction of eight peptides named Pep1-Pep8. Four of these peptides were constructed linearly considering conserved structural elements such as the N-terminal region (Pep1) and loop 2 (Pep2-Pep4). In the mimetic peptides, the three conserved regions of cystatins - N-terminal region, loop 1, and loop 2 - are considered.

**Figure 6.**
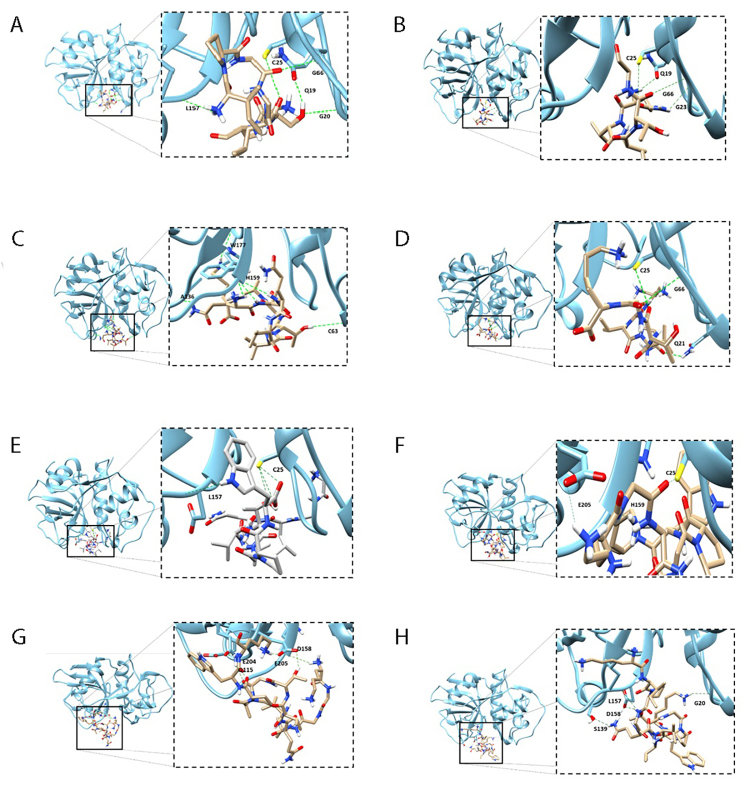
Graphical representation of the *in silico* interaction of cruzain (blue) with peptides derived from the main structure of AsCystatin. The eight designed peptides interact through hydrogen bonds with key amino acid residues of the enzyme, including those in the catalytic site as well as those located in the S2 and S3 subsites.

A detailed analysis of the primary structure of AsCystatin enabled the design of eight peptides (Pep1-Pep8). Figure 5 presents a schematic representation of the eight designed peptides, with four of them (Pep1 to Pep4) being constructed linearly, considering conserved structural elements of cystatins, such as glycine in the N-terminal region (Pep1) and loop 2 (Pep2 to Pep4), while Pep5 to Pep8, termed mimetic peptides, include the N-terminal region, loop 1, and loop 2. Molecular docking reveals that all constructed peptides can interact *in silico* with key regions of cruzain (Figures 6 A to 6H and Table 2).

**Table 2.** Sequence, molecular mass, and interaction details of the peptides derived from AsCystatin with *T. cruzi* cruzain.

Peptide	Sequence	MM (Da)	Amino acid residues involved in protein-protein interaction	Interaction energy (kcal/mol)
Pep1	IPGGLSK	670.81	Q19, G20 C25, G66, L157	-5.1
Pep2	QVVTG	502.57	Q19, C25, G23, G66	-6.6
Pep3	QSQVVTGAK	917.03	A36, C63, H159, W177	-6
Pep4	KQVVTGK	752.92	Q21, C25, G66	-5.5
Pep5	GGLQVVTGPW	1.0131	C25, L157	-6.8
Pep6	KGGLQVVTGPWK	1.2695	C25, H159, E205	-7.7
Pep7	KGGLQVVTGAPWK	1.3405	Q115, D158, H159 E204, E205	-8.1
Pep8	KIPGGQVVTGPWK	1.3366	G20, S139, L157, D158	-6.7
Interaction control	MMTS		C25	-7.4

### In vitro inhibitory potential of peptides derived from AsCystatin on cruzain enzymatic activity

The eight designed peptides were synthesized and assessed for their *in vitro* inhibitory potential against *T. cruzi* protease. *In vitro* assays demonstrated that only four designed mimetic peptides significantly inhibited cruzain (Table 3).

**Table 3.** Inhibitory potential of AsCystatin-derived peptides on cruzain proteolytic activity.

Peptide	Sequence	I_50_(µM) ± SD
Pep1	IPGGLSK	> 500
Pep2	QVVTG	> 500
Pep3	QSQVVTGAK	> 500
Pep4	KQVVTGK	> 500
Pep5	GGLQVVTGPW	220 ± 0.48
Pep6	KGGLQVVTGPWK	384.6 ± 0.77
Pep7	KGGLQVVTGAPWK	388.5 ± 1.22
Pep8	KIPGGQVVTGPWK	281.5 ± 2.19

### In vitro trypanocidal and cytotoxic activity of synthesized peptides

Using epimastigote forms of *T. cruzi* as a model, three of the four cruzain inhibitory mimetic peptides exhibited trypanocidal activity *in vitro* against the parasites (Table 4 and Additional file 1). In contrast, none of the peptides showed any cytotoxic effect at the highest assessed concentration on murine fibroblasts of the NCTC929 lineage (CC50 > 500 (M). 

**Table 4.** In vitro trypanocidal and cytotoxic activity of AsCystatin-derived peptides using epimastigote forms of T. cruzi and NCTC929 cells, respectively.

Peptide	Sequence	IC_50_ (µM) ± SD	IC_50_ (µM) ± SD	SI
Pep1	IPGGLSK	> 500	> 500	ND
Pep2	QVVTG	> 500	> 500	ND
Pep3	QSQVVTGAK	> 500	> 500	ND
Pep4	KQVVTGK	> 500	> 500	ND
Pep5	GGLQVVTGPW	475.5 ± 1.65	> 500	> 1.05
Pep6	KGGLQVVTGPWK	> 500	> 500	ND
Pep7	KGGLQVVTGAPWK	390.2 ± 2.1	> 500	> 1.28
Pep8	KIPGGQVVTGPWK	471.3 ± 1.8	> 500	> 1.06

ND: not determined; SI: selectivity index.

## DISCUSSION

As mentioned above, despite over a century having passed since the discovery of Chagas disease, there are several reasons why this disease continues to have a severe impact on global public health. The limited treatment efficacy in the chronic phase of the disease, side effects, and other factors highlight the need for research to identify new molecules to assist current chemotherapy [37, 38]. Studies proposing molecular targets as tools for the search for potential inhibitors of key molecules for the parasite have emerged as promising strategies for identifying trypanocidal agents [9-11]. Given the importance of cruzain in critical stages of the *T. cruzi* life cycle, this study aimed to explore the *in vitro* inhibitory potential of a recombinant cystatin from the venom of *A. superbus* and its derived peptides, as well as assess the *in vitro* trypanocidal activity of the peptides.

Initially, considering the advantages of *E. coli* as a model organism for recombinant protein production, the BL21 DE3 strain of this bacterium was used for cruzain production. Notably, over the years, experimental stages for obtaining recombinant cruzain have undergone various modifications optimizing the process [12, 39-41]. In this regard, we opted to use the methodology proposed by Lee et al. [31], introducing some specific modifications. The main modification was using 1 mM IPTG as an inducer for protein production, maintaining the same time and temperature conditions proposed by Lee et al. [31], which allowed for the slow and gradual expression of pro-cruzain. This factor contributed to obtaining the zymogen in a soluble form. Figure 1 A shows the electrophoretic profile of the lysate of transformed bacteria before IPTG addition (lane 1) and 72 hours after induction (lane 2), where a prominent band with proteins of approximately 35 kDa, compatible with pro-cruzain, was observed.

Subsequently, the bacterial lysate was subjected to affinity chromatography for pro-enzyme purification. Figure 1 B shows the chromatographic profile of the protein, which was eluted with approximately 35% elution buffer. The electrophoretic profile in Figure 1 C (lane 1) presents a prominent band of proteins with a molecular weight of 35 kDa, compatible with pro-cruzain. A fainter band of approximately 23 kDa, corresponding to mature cruzain, can also be observed in the same sample. The presence of this band may be due to the onset of auto-proteolysis, which was interrupted by adding protease inhibitors to the product.

As previously reported, in order to obtain proteins in higher concentrations and with a higher degree of purity, the steps for the production of recombinant cruzain have undergone changes. As mentioned by Lee et al [31], the previously reported methodologies [12, 39] allowed obtaining this protease in the insoluble fraction, which led to further denaturation steps using urea, renaturation of the proteins and dialysis steps, which resulted in a lower concentration of active protein at the end of the process. The protocol used as a model in this study allowed obtaining high levels of pro-cruzain, thus avoiding denaturation and refolding steps. Moreover, the presence of the histidine tail in the expressed protein allowed the purification of pro-cruzain by IMAC chromatography with a high degree of solving. In this way, the protein was already eluted in the correct folding, and the process of activation in its catalytic form by autoproteolysis was carried out in a much shorter time than that reported in the previous protocols. In this study, it was possible to obtain approximately 15 mg of active protein per liter of induced culture, a concentration considerably lower than that obtained in the protocol used as a model, in which they reported approximately 60 mg/L, which may be due to the changes introduced in the protocol belonging to this study. It is important to note that although a lower yield was observed compared to the amount of recombinant protein obtained in the model protocol, this decrease did not affect the development of subsequent experiments. Another important consideration is that to prevent cruzain from undergoing auto-proteolysis, it had to be expressed as a zymogen devoid of enzymatic activity. Once the pro-form was purified, the enzyme activation process was carried out.

To initiate activation by auto-proteolysis, pro-cruzain was subjected to dialysis and then incubated in its activation buffer. Subsequently, 5 mM DTT was added to remove the MMTS inhibitor, covalently bound to the cysteine residue in the enzyme's catalytic triad. Figure 1 D shows that the protein remains in its zymogen form for up to 20 minutes, while from 40 minutes onward, only the mature protease is observable. A band of proteins with a molecular weight of approximately 13 kDa, corresponding to the pro-domain cleaved by the auto-proteolysis process, is also visible. The procedure demonstrated that from 40 to 120 minutes, mature cruzain could be obtained in a soluble, enzymatically active form with appropriate purity and concentration to proceed with subsequent experiments.

As mentioned earlier, one of the objectives of this study was to identify cruzain inhibitors. To better understand the rationale behind this study, it is important to highlight three key premises: (i) cruzain is a cysteine protease belonging to the same family as papain and various mammalian cathepsins; (ii) the high structural and functional similarity between this group of proteins and cathepsins L and B [42-44]; and (iii) the inhibitory effect of cystatins on the enzymatic activity of papain and cathepsins [45-48]. Based on this, the hypothesis was proposed that cystatins present in the venom of snakes could also act as potential cruzain inhibitors. Based on the work of Richards et al. [33], it was decided to carry out the recombinant expression of the cystatin from *A. superbus* venom.

Before this, preliminary assessments were conducted using bioinformatics tools to assess the possible interactions with cruzain and characterize the cystatin as an inhibitor. These processes included homology modeling of the cystatin and an *in silico* interaction assay between the drug target and its potential inhibitor.

The first step was to develop the three-dimensional model of the cystatin, using the protein with PDB code 4N60, corresponding to human cystatin M, as a template. Figure 2 shows that AsCystatin exhibits tertiary structural elements characteristic of group 2a cystatins, such as the "cystatin fold", consisting of five antiparallel β-strands surrounding a central α-helix [45, 49, 50]. Additionally, other conserved regions were identified, such as (i) the N-terminal region, characterized by a glycine residue at position 11; (ii) "loop 1," formed by residues QxVxGm; (iii) "loop 2," formed by residues PW; and (iv) a site termed "back side loop" (BSL), situated between the central α-helix and the second β-strand [33, 45-51]. Subsequently, the *in silico* molecular interaction between the three-dimensional model of AsCystatin and cruzain was assessed. Figure 2 B shows the details of the interaction between both proteins, revealing that key residues of cruzain are involved in this interaction. Cys25 and His159, belonging to the catalytic triad, interact with residues from loop 1 of AsCystatin. Similarly, Leu157 and Asp158, which belong to subsites S2 and S3 of cruzain, are also involved in the interaction [39, 52]. The results obtained from *in silico* interaction studies, combined with the inhibitory potential of cystatins on cysteine proteases, suggest that AsCystatin could act as an inhibitor of cruzain. The next step was the recombinant production of AsCystatin.

Considering the biotechnological potential of cystatins [51, 53, 54], various research groups have developed protocols to optimize the recombinant production of these proteins [55-58], focusing primarily on aspects related to solubility and yield. In this study, the expression of the potential inhibitor was approached using the protocol proposed by Richards et al. [33], with some modifications, such as gene optimization and the insertion of a histidine tag at the C-terminal region. These changes affected the purification process of the recombinant product compared to the original protocol. Different concentrations of the inducer and different induction times and temperatures were also assessed.

Initially, induction was carried out with 0.1 and 0.5 mM IPTG for 16 hours at 16°C. Figure 3 A shows the electrophoretic profile of the bacterial culture before (lane 2) and after induction (lanes 1 and 3). Bands corresponding to proteins with an approximate molecular mass of 13 kDa were observed, consistent with the molecular mass of cystatins, suggesting the expression of the protein of interest. The induced cultures were then subjected to affinity chromatography, and Figures 3 B and 3C show that proteins under both induction conditions were purified with approximately 30% elution buffer. Figure 3 D presents the electrophoretic profile of the purified proteins, demonstrating that under the assessed conditions, AsCystatin was obtained in a soluble form with a high degree of purity. Additionally, it was observed that increasing the IPTG concentration to 1 mM and changes in induction times and temperatures did not favor the soluble expression of the protein (data not shown). Therefore, it is suggested that the most appropriate condition for obtaining soluble AsCystatin is induction with 0.1 mM IPTG for 16 hours at 16°C.

Once both proteins were obtained, the inhibitory potential of AsCystatin on cruzain was assessed. In this experiment, it was observed that most of the assessed concentrations of AsCystatin significantly reduced the enzymatic activity of the target molecule (Figure 4), with a concentration of 21.2 µM needed to inhibit 50% of cruzain's activity (Table 1). It is worth noting that literature reports that cystatins isolated from various sources can competitively bind to cysteine proteases, causing inhibition of these enzymes. Regarding cystatins from family 2, it is suggested that amino acid residues present in the N-terminal region and those in loop 1 and 2 are key for interaction and inhibition of proteases [46, 47, 51, 55, 59, 60]. Similarly, cystatins have been reported as interesting models for designing cruzain inhibitors, suggesting that these proteins are promising molecules for potentially inhibiting *T. cruzi* protease [13, 61].

Subsequently, once the potential of AsCystatin as an inhibitor was confirmed, an analysis of the primary structure of this protein was performed. As reported by Richards et al. [33], conserved regions in cystatins responsible for interaction with cysteine proteases include Glycine in the N-terminal region, loop 1, formed by QVVTG, and residues P and W in loop 2. Various authors have suggested that peptides derived from cystatins with sequence identity to the consensus regions mentioned can act as cysteine protease inhibitors [62-67]. In this context, linear and mimetic peptides derived from the primary structure of AsCystatin were proposed (Figure 5) to initially assess through *in silico* assays whether the designed peptides could interact with the protease and subsequently perform *in vitro* enzyme inhibition assays and finally determine their trypanocidal and cytotoxic potential in cell cultures.

Before synthesizing the peptides, *in silico* interaction analyses between cruzain and the designed peptides were conducted (Table 2 and Figures 6 A to 6H). These results suggest that all 8 peptides interact with crucial regions of cruzain, including both the catalytic site and residues in subsites S2 and S3. Regarding peptide design, peptides 1 to 4 were designed linearly, considering conserved structural elements in the N-terminal region and loop 1 of cystatins. Pep1 includes (as described in Materials and Methods) the first 7 residues of the N-terminal region, including the conserved glycine residue. Pep2 consists solely of the consensus sequence QVVTG from loop 1, while Pep3 includes the consensus sequence with two additional amino acids at both ends (Figure 5). Finally, Pep4 had lysine residues added to each end to provide a more positive charge to these peptides. According to *in silico* analyses, these peptides interact with Cys25 and His159 of the catalytic site of cruzain.

For the design of the remaining 4 peptides, the strategy proposed by Lamanach et al. [66] was followed, which involved constructing mimetic peptides. These peptides were designed with residues from the N-terminal region and loop 1 and 2, responsible for interacting with cysteine proteases. *In silico* analyses suggest that the 4 mimetic peptides interact with cruzain in both the catalytic site and residues corresponding to subsites. It is important to note that lysine residues were added to both ends of peptides 6, 7, and 8 to provide a more significant positive charge and improve solubility. Additionally, mimetic peptides showed lower interaction energy than linear peptides, possibly due to their higher positive charge, which could favor electrostatic interactions considering the negative isoelectric point of cruzain. The docking results presented in this study were obtained through a rigorous and systematic selection process designed to identify peptide models with optimal binding energies and interaction profiles. For each peptide, multiple docking poses were generated and thoroughly analyzed to ensure that the final models represented the most favorable binding interactions with cruzain's catalytic triad (Cys25, His159, and Asp158). This meticulous approach ensured that the selected models accurately reflect the peptides' inhibitory potential, avoiding reliance on the first generated poses. By prioritizing poses with the lowest binding energies and strongest interactions, this study provides robust insights into the structure-activity relationships of the peptides and their predicted biological efficacy.

In this context, and based on the *in silico* results, all eight peptides were synthesized, and their inhibitory potential on cruzain was assessed. Table 3 shows that at the highest concentration tested, linear peptides assessed in this study did not exhibit inhibitory activity on cruzain. In contrast, the mimetic peptides significantly reduced the enzymatic activity of the protease. Notably, despite literature reporting potential inhibitory effects of linear peptides [63, 64, 66], particularly those with the QxVXG sequence, the linear peptides assessed in this study did not inhibit cruzain. On the other hand, mimetic peptides demonstrated inhibitory effects on the protease, suggesting that all three consensus regions are necessary for achieving inhibitory effects. Additionally, adding lysine residues to the mimetic peptides improved their solubility and increased their positive charge, which could be a key factor contributing to enzyme interaction and inhibition. 

Finally, using *T. cruzi* epimastigotes and NCTC 929 mouse fibroblasts as models, the trypanocidal and cytotoxic potential of the 8 synthetic peptides was assessed. It was observed that 3 out of the 4 mimetic peptides exhibited trypanocidal effects (Table 4), while none of the assessed peptides showed cytotoxic effects on mouse fibroblasts. It should be highlighted that snake venom-derived peptides have been explored as promising tools for identifying antimicrobial agents [68-70]. Although cystatins and peptide fragments from these inhibitors have been assessed for their microbicidal potential, reporting bactericidal [71], antifungal [72], and antiparasitic [73] activities, the results obtained in this study using peptides derived from a snake cystatin as a model for identifying trypanocidal agents are novel. 

 In this study, Pep7 demonstrated the highest overall efficacy among the peptides tested, a distinction that can be attributed to its superior binding energy and well-defined structure-activity relationship. With a predicted binding energy of −8.1 kcal/mol, Pep7 exhibited highly favorable interactions with cruzain's catalytic and allosteric sites, as evidenced by molecular docking analyses. These interactions included hydrogen bonds with key residues in cruzain's catalytic triad, which are essential for enzymatic activity, along with additional stabilization through van der Waals forces. The alignment of these interactions with cruzain’s active site geometry suggests that Pep7's design effectively targets critical regions of the enzyme, maximizing binding strength and specificity.

The robust binding properties of Pep7 were corroborated by its performance in both enzymatic inhibition and antiparasitic assays. Pep7 consistently demonstrated consistent IC50 values in the proteolytic and trypanocidal assays, underscoring its superior inhibitory capacity. This dual efficacy indicates that Pep7 not only disrupts cruzain’s enzymatic activity with high specificity but also translates this inhibition into a measurable reduction in parasite viability. The agreement between its predicted binding energy and experimental results highlights the predictive accuracy of the molecular docking approach employed, as well as the peptide’s structural optimization. 

It is important to highlight that although the IC50 values achieved with the three peptides with trypanocidal activity can be considered high concentrations, these findings are promising, because these identified molecules could be used as a model to improve the construction of new structures that are able to optimize the antiparasitic activity. Given the observed results, further studies are suggested to improve the inhibitory effect on cruzain activity and, consequently, the antiparasitic potential against *T. cruzi* of the molecules under study.

## Conclusion

There is an urgent need to develop research to identify molecules that could contribute to the chemotherapy of Chagas disease. Given the functional importance of cruzain for *T. cruzi*, this cysteine protease presents itself as an interesting target for inhibitor discovery. In search of inhibitors, AsCystatin showed inhibition capacity over cruzain. Four AsCystatin-derived mimetic peptides showed inhibitory activity without cytotoxic effects in mouse fibroblasts. Based on the results obtained in this study, it is necessary to continue with structural and functional studies of peptides derived from cystatins, as these can help optimize the inhibitory and trypanocidal potential of these molecules, making them promising tools for the development of new trypanocidal agents. 

## Supplementary material

The following online material is available for this article:

Additional file 1 - Sigmoid dose-response curve of: **(A)** trypanocidal activity of the reference drug benznidazole on epimastigote forms of *T. cruzi* and **(B)** cytotoxic activity on mammalian cells. **(C)** Typanocidal activity of the active peptides.
