# Supplementary material for: Cystatin from Austrelaps superbus snake venom as a model for identifying potential inhibitors of Trypanosoma cruzi cruzain
Source: J Venom Anim Toxins Incl Trop Dis. 2025 Feb 14;31:e20240055. doi: 10.1590/1678-9199-JVATITD-2024-0055 (PMC11832194; doi:10.1590/1678-9199-JVATITD-2024-0055)
Supplement: Additional file 1 - [file 1678-9199-jvatitd-31-e20240055-s1.pdf]

J Venom Anim Toxins incl Trop Dis

https://doi.org/10.1590/1678-9199-JVATITD-2024-0055

Supplementary Material to "Cystatin from *Austrelaps superbus* snake venom as a model for identifying potential inhibitors of *Trypanosoma cruzi* cruzain"

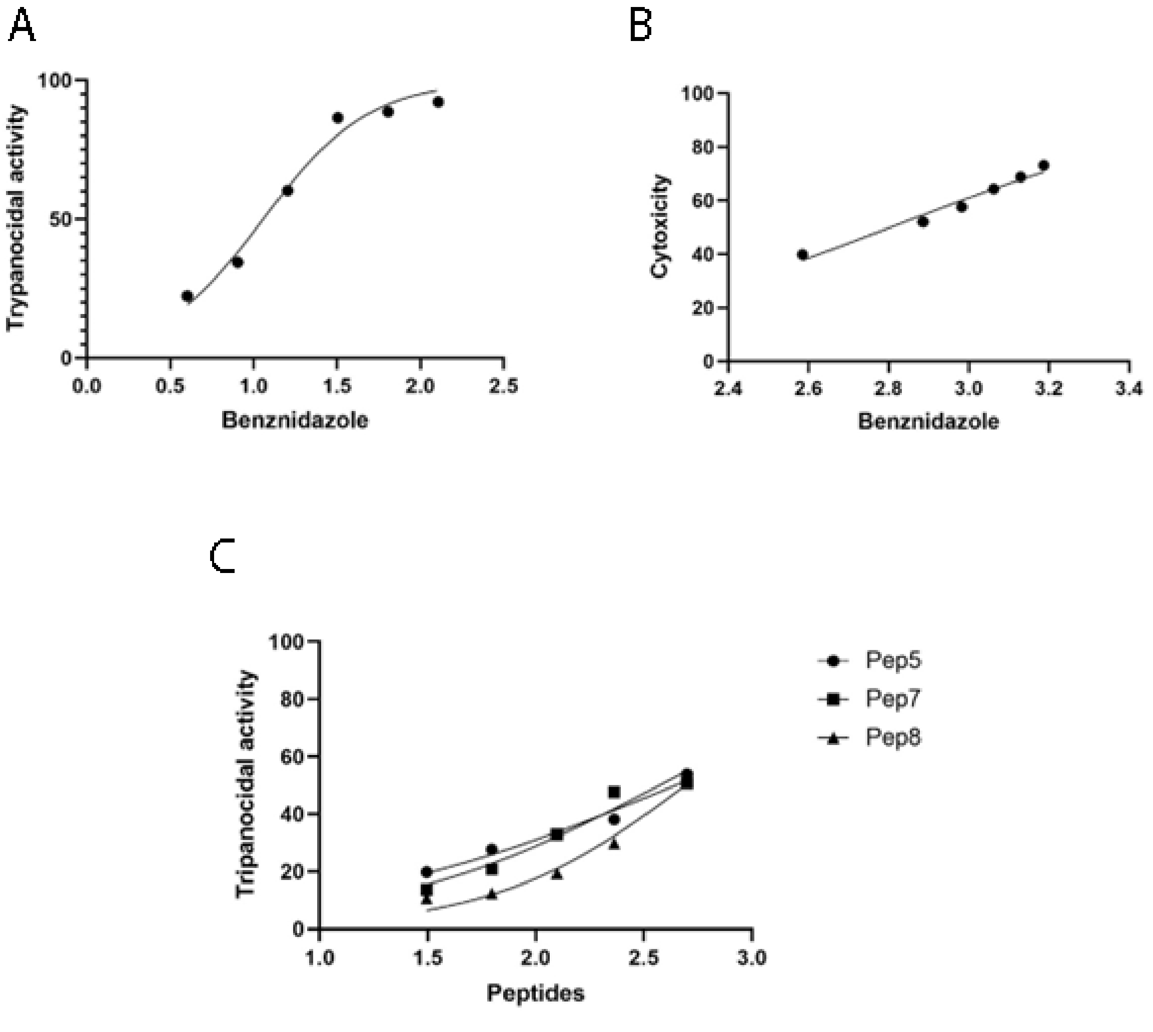

Additional file 1. Sigmoid dose-response curve of: **(A)** trypanocidal activity of the reference drug benznidazole on epimastigote forms of *T. cruzi* and **(B)** cytotoxic activity on mammalian cells. **(C)** Typanocidal activity of the active peptides.
